# The antimicrobial potential of *Streptomyces* from insect microbiomes

**DOI:** 10.1038/s41467-019-08438-0

**Published:** 2019-01-31

**Authors:** Marc G. Chevrette, Caitlin M. Carlson, Humberto E. Ortega, Chris Thomas, Gene E. Ananiev, Kenneth J. Barns, Adam J. Book, Julian Cagnazzo, Camila Carlos, Will Flanigan, Kirk J. Grubbs, Heidi A. Horn, F. Michael Hoffmann, Jonathan L. Klassen, Jennifer J. Knack, Gina R. Lewin, Bradon R. McDonald, Laura Muller, Weilan G. P. Melo, Adrián A. Pinto-Tomás, Amber Schmitz, Evelyn Wendt-Pienkowski, Scott Wildman, Miao Zhao, Fan Zhang, Tim S. Bugni, David R. Andes, Monica T. Pupo, Cameron R. Currie

**Affiliations:** 1 Laboratory of Genetics, University of Wisconsin-Madison, Madison, 53706 WI USA; 2 Department of Bacteriology, University of Wisconsin-Madison, Madison, 53706 WI USA; 3 School of Pharmaceutical Sciences of Ribeirão Preto, University of São Paulo, Ribeirão Preto, 14040-903 SP Brazil; 4 Pharmaceutical Sciences Division, School of Pharmacy, University of Wisconsin-Madison, Madison, 53705 WI USA; 5 McArdle Laboratory for Cancer Research, Wisconsin Institute for Medical Research, University of Wisconsin-Madison, Madison, 53705 WI USA; 6 Department of Molecular and Cell Biology, University of Connecticut, Storrs, 06269 CT USA; 7 Department of Biology, Large Lakes Observatory, University of Minnesota-Duluth, Duluth, 55812 MN USA; 8 School of Biological Sciences, Georgia Institute of Technology, Atlanta, 30332 GA USA; 9 Center for Research in Microscopic Structures and Department of Biochemistry, School of Medicine, University of Costa Rica, San José, 10102 Costa Rica; 10 Department of Medicine, University of Wisconsin School of Medicine and Public Health, Madison, 53705 WI USA

## Abstract

Antimicrobial resistance is a global health crisis and few novel antimicrobials have been discovered in recent decades. Natural products, particularly from *Streptomyces*, are the source of most antimicrobials, yet discovery campaigns focusing on *Streptomyces* from the soil largely rediscover known compounds. Investigation of understudied and symbiotic sources has seen some success, yet no studies have systematically explored microbiomes for antimicrobials. Here we assess the distinct evolutionary lineages of *Streptomyces* from insect microbiomes as a source of new antimicrobials through large-scale isolations, bioactivity assays, genomics, metabolomics, and in vivo infection models. Insect-associated *Streptomyces* inhibit antimicrobial-resistant pathogens more than soil *Streptomyces*. Genomics and metabolomics reveal their diverse biosynthetic capabilities. Further, we describe cyphomycin, a new molecule active against multidrug resistant fungal pathogens. The evolutionary trajectories of *Streptomyces* from the insect microbiome influence their biosynthetic potential and ability to inhibit resistant pathogens, supporting the promise of this source in augmenting future antimicrobial discovery.

## Abstract (web summary)

Host microbiomes are feasible sources for drug discovery. Here, using large-scale isolations, bioactivity assays and omics, the authors uncover the antimicrobial potential of insect-associated *Streptomyces* and identify a compound, cyphomycin, active against multidrug-resistant fungal pathogens.

## Introduction

The rapid emergence of antimicrobial resistance in bacterial and fungal pathogens is a public health crisis^1,2^. Novel therapeutics are needed to counter resistance, yet no new antimicrobial classes have been clinically approved in over three decades^3^. Natural products are the main source of antimicrobials, the majority of which are produced by Actinobacteria cultured from the soil^3,4^. However, contemporary studies of this once prolific source of novel chemistry face dramatically diminishing returns, largely due to the rediscovery of known compounds^5^. Efforts to address this issue, including genome mining^6,7^, synthetic biology^8^, and exploring alternative microbial sources, such as marine microbial environments^9–12^ and underrepresented taxa^13,14^, have yielded limited success. To combat the continual emergence of multidrug-resistant pathogens, there is a critical and constant need to discover new antimicrobial natural products.

**Fig. 1:**
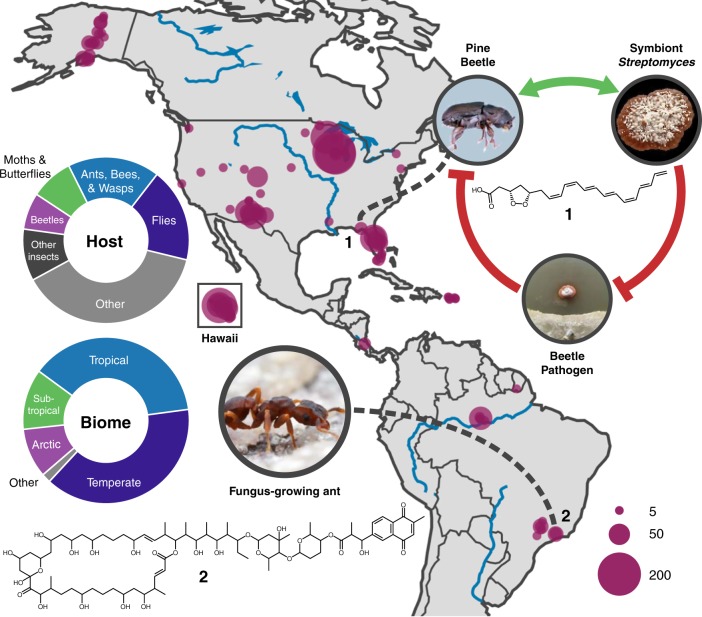
Sampling strategy for *Streptomyces* from insect microbiomes. *Streptomyces* were isolated from a wide range of insects and geographies (1445 insects; 10,178 strains; dot size, insects sampled). *Streptomyces* production of the antifungal mycangimycin (**1**) in the Southern Pine Beetle system is shown at right. Cyphomycin (**2**) is a new antifungal described herein. Photo credits: southern pine beetle - Erich G. Vallery; fungus-growing ant – Alexander Wild

Natural products are the language of microbial interactions, evolved to mediate communication and antagonism among and between species^15,16^. Within microbiomes, the ecology and diversity of natural product chemistry reflect the underlying interactions between the microbial community, host, and environment. Exploration of the specialized natural product chemistry embedded within host microbiomes is an emerging new paradigm in antimicrobial drug discovery. Antimicrobials have recently been discovered from the microbiomes of diverse eukaryotic hosts, ranging from sea squirts^17^ to humans^18^. A particularly compelling source of novel antimicrobials lies in defensive symbioses, where bacterial symbionts produce antimicrobials to protect against opportunistic and specialized pathogens^19–23^. In insects, these symbioses are best exemplified in fungus-growing ant^19–21^, solitary digger wasp^22^, and southern pine beetle^23^ (Fig. 1a, right) systems, where Actinobacteria (typically *Streptomyces*) provide chemical defenses, paralleling our own reliance on the antimicrobials produced by these taxa to combat infectious disease. For example, the *Streptomyces* symbiotically associated with the southern pine beetle (*Dendroctonus frontalis*) produce the secondary metabolites frontalamide A, frontalamide B, and mycangimycin^23,24^. Mycangimycin inhibits the beetles’ antagonistic fungus *Ophiostoma minus* and has potent inhibitory activity against malaria while the frontalamides have general antifungal activity^23,24^. Solitary wasps also associate with *Streptomyces* that provide antibacterial and antifungal chemical protection to their larvae through production of streptochlorin, a variety of piericidin analogs, and other molecules^25^. The antifungal compound sceliphrolactam was discovered from *Streptomyces* associated with a mud dauber wasp^25^. The natalamycin derivatives produced by *Streptomyces* from the fungus-growing termite system provide similar antifungal defense^26^. Further, over 10 new natural products with antimicrobial activity have been identified from the chemical characterization of approximately 100 insect-*Streptomyces* strains^23,24,27,28^. Globally there are over five million insect species that occupy virtually every terrestrial niche^29^. Although insects are among the most diverse organisms on the planet^29^, studies of Actinobacteria from these systems have been limited to only a few insect orders, specifically Hymenoptera and Coleoptera. Further, insects themselves exhibit complex chemistry that mediates and maintains the diversity of their ecological interactions^30^.

Here, we systematically examine our hypothesis that insect microbiomes are a valuable source of new antimicrobials. The extreme diversity of insects presents untapped potential for drug discovery from their equally diverse microbial communities. However, the breadth of natural product biosynthesis and antimicrobial potential within insect microbiomes remains relatively unknown. We hypothesize that *Streptomyces* from insect microbiomes represent a promising source of antimicrobials with distinct evolutionary histories from soil *Streptomyces*, upon which most antimicrobial discovery efforts have focused. We focus on *Streptomyces* because this genus: (i) is the source of most clinically used antibacterials and antifungals, (ii) has established genetic tools to facilitate development^8^, and (iii) has been implicated in readily forming associations with diverse insect hosts^31^.

## Results

### *Streptomyces* are commonly found in insect microbiomes

Counter to the prevailing assumption of *Streptomyces* being largely soil-associated bacteria, in our shotgun metagenome analyses, *Streptomyces* comparably occur in host-associated and soil-associated contexts. Specifically, we calculate the number of *Streptomyces* reads per megabase (rpM) to be 129.32 rpM and 172.72 rpM for host-associated and soil studies, respectively (data from ref. ^4^). Metagenomes from freshwater (47.49 rpM) and marine (24.65 rpM) sources are much lower in *Streptomyces* abundance and support the hypothesis that most *Streptomyces* are either soil- or host-associated.

**Fig. 2:**
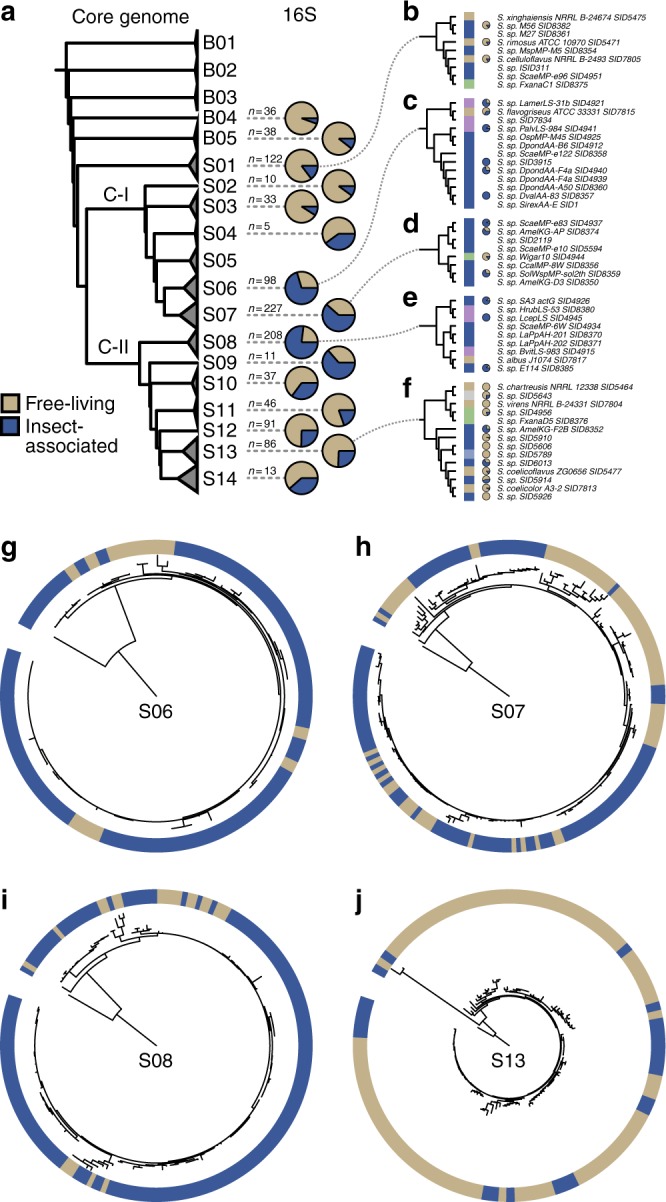
Distinct lineages of *Streptomyces* associate with insect hosts. **a** A genomic phylogeny constructed from 93 single-copy core bacterial genes is shown to the left with the major clades, Clade I (C–I) and Clade II (C-II), labeled (B = Basal, S = *Streptomyces*). 16S sequences were mapped to the genomic phylogeny and the distribution of free-living (tan) and insect-associated (blue) strains is shown as both pie and bar charts to the right. Number of total strains is shown to left of pies. **b**–**f** A more detailed mapping of 16S sequences onto the genomic tree is shown for clades S01 (**b**), S06 (**c**), S07 (**d**), S08 (**e**), and S13 (**f**). **g**–**j** 16S phylogenies from sequences mapped to clade S06 (**g**), S07 (**h**), S08 (**i**), and S13 (**j**)

Focusing on insect hosts, we first determined that associations between *Streptomyces* and insects are widespread through sampling 2561 insects spanning 15 taxonomic orders (Fig. 1, host donut chart, and Supplementary Data 1) and a wide range of geographies and biomes (Fig. 1, map and biome donut chart). Actinobacteria were isolated from 1445 of 2580 insect microbiomes (56%) spanning 13 orders, resulting in 10,178 individual isolates, including 2934 from Hymenoptera, 2920 from Diptera, 1326 from Lepidoptera, and 1139 from Coleoptera (Supplementary Data 2). Additionally, 6935 isolates were obtained from other sources (soil: *n* = 833; plants: *n* = 980). Phylogenetic placement of 536 insect-associated and 571 free-living strains indicates that specific lineages of *Streptomyces* are enriched for associations with insects (Fig. 2).

### Insect-*Streptomyces* exhibit high inhibitory activity

**Fig. 3:**
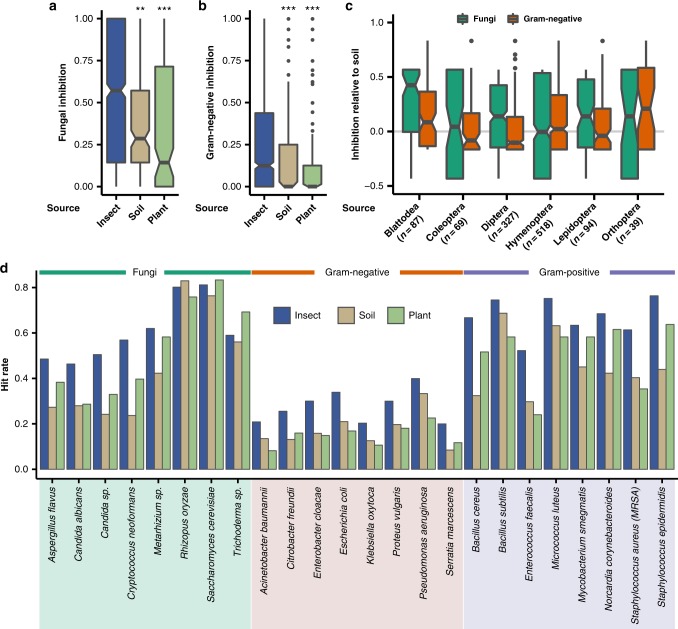
Bioactivity of insect-associated *Streptomyces*. **a** Fungal and **b** Gram-negative pathogens are significantly more inhibited by insect-associated isolates compared to soil- and plant-sourced *Streptomyces* (*n* = 1162, 186, and 178 for insect, soil, and plant, respectively; ***p < 1e−3; **p < 1e−2; *t*-test, BY correction). **c** Strains vary in antimicrobial bioactivity by insect host orders (*n* = 87, 69, 327, 518, 94, and 39 for Blattodea, Coleoptera, Diptera, Hymenoptera, Lepidoptera, and Orthoptera, respectively). **a**–**c**: center, median; box, upper and lower quantiles; notches, 95% confidence; whiskers, 1.5× interquartile range; points, outliers. **d** Hit rate for insect, soil, and plant strains against individual pathogens (*n* = 1162, 186, and 178 for insect, soil, and plant, respectively)

Through 51,050 individual antimicrobial bioactivity assays, pairing 2003 *Streptomyces* strains against a panel of 27 clinically and/or ecologically relevant microbes (Supplementary Data 3), we show that insect-associated strains (*n* = 1162) exhibited significantly greater inhibitory activity towards fungi, Gram-negative bacteria, and Gram-positive bacteria, compared to both soil (*n* = 186) and plant-associated (*n* = 178) *Streptomyces* isolates (Fig. 3a–c, Supplementary Figure 1A, and Supplementary Data 4). Specifically, insect *Streptomyces* strains had significantly greater antifungal activity, as indicated by inhibition fractions, the fraction of fungi for which a strain exhibited antimicrobial inhibition, where average inhibition fractions for insect-*Streptomyces* were 0.52 ± 0.01 fraction inhibition compared to 0.42 ± 0.02 (*p* = 9.46e−3; *t*-test, BY correction) and 0.36 ± 0.03 (*p* = 7.84e−7; *t*-test, BY correction) for soil and plant-associated strains, respectively (Fig. 3a). Against Gram-negative bacteria, insect-associated strains had an inhibition fraction of 0.26 ± 0.01 compared to 0.17 ± 0.02 (*p* = 6.45e−4; *t*-test, BY correction) and 0.12 ± 0.02 (*p* = 2.07e−7; *t*-test, BY correction) for soil and plant-associated *Streptomyces*, respectively (Fig. 3b). Against Gram-positive bacteria, insect-associated strains had an inhibition fraction of 0.61 ± 0.01 compared to 0.45 ± 0.02 (*p* = 7.84e—7; *t*-test, BY correction) and 0.34 ± 0.03 (*p* = 7.06e−16; *t*-test, BY correction) for soil and plant-associated strains, respectively (Supplementary Figure 1A). Some insect host orders associate with *Streptomyces* with especially high activity against Gram-negative bacteria and fungi (Fig. 3c). For example, strains isolated from Orthoptera (crickets and grasshoppers; *n* = 39), Blattodea (termites and cockroaches; *n* = 87), and Hymenoptera (ants, bees, and wasps; *n* = 518) had particularly high inhibition against Gram-negative bacteria and had inhibition fractions of 0.25, 0.16, 0.11 higher than the average soil isolate, respectively. In challenges against fungi, strains from Blattodea (termites and cockroaches; *n* = 87) and Lepidoptera (moths and butterflies; *n* = 94) had particularly effective antimicrobial activity, with inhibition fractions greater than that of the average soil isolate by 0.26 and 0.13, respectively. Similar trends were seen in Gram-positive assays (Supplementary Figure 1B).

Insect-associated strains have higher bioactivity against these pathogen classes compared to soil Actinobacteria (Fig. 3a–c, Supplementary Figure 1A-B) and variation of activity between host orders suggests the microbiomes of some insect lineages are better equipped to defend against specific pathogen classes (Fig. 3c, Supplementary Figure 1D-E). Furthermore, insect-associated *Streptomyces* generally have higher rates of inhibition (i.e., hit rate) against many important clinical pathogens, including *Pseudomonas aeruginosa*, *Acinetobacter baumannii*, *Candida albicans*, and methicillin-resistant *Staphylococcus aureus* (Fig. 3d). These assays reflect traditional, albeit high-throughput, approaches for antimicrobial screening. Biosynthesis of secondary metabolites is often dependent on many factors not present in the lab, so metabolites from certain environments (e.g., a host-associated *Streptomyces* cultured in the absence of environmental cue) may not be produced under these conditions^32,33^. Nevertheless, significantly higher overall inhibitory activity of insect *Streptomyces* compared to soil strains suggests insects generally associate with strains with greater antimicrobial potential and thus represent a promising source for antimicrobial discovery.

### Biosynthetic potential is shaped by ecology and phylogeny

**Fig. 4:**
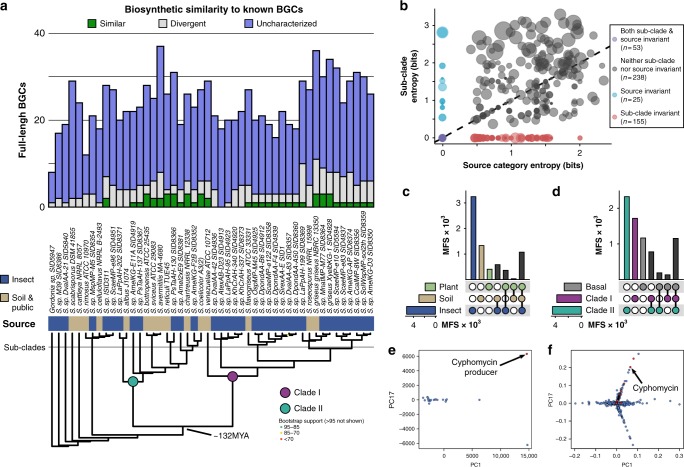
Ecology and phylogeny influence biosynthetic potential. **a** A core-genome phylogeny shows evolutionarily distinct lineages of *Streptomyces* associate with insects (subset shown, see Supplementary Figure 2). BGC similarity to known BGCs highlights the biosynthetic diversity of insect microbiome strains. **b** Source invariant (blue) and sub-clade invariant (red) BGC families suggest BGC presence is influenced by both source and phylogeny. LC/MS metabolomics revealed MFs that are unique to **c** source and **d** phylogeny. **e** PCA of the metabolomes identified an outlier strain and **f** MFs that contribute to its uniqueness, including cyphomycin

**Fig. 5:**
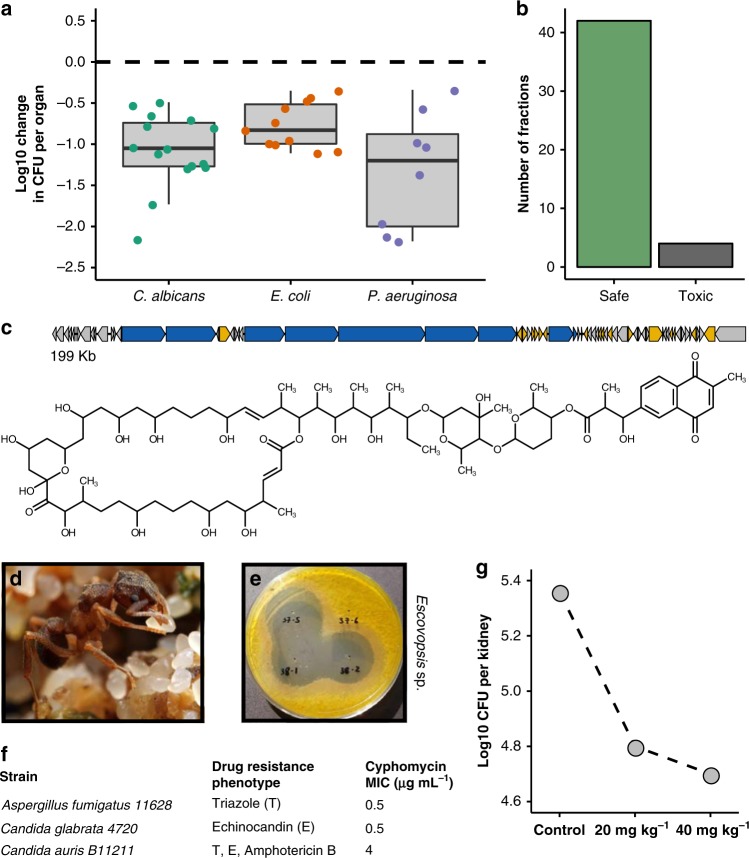
Insect-associated *Streptomyces* are a source of active antimicrobials. **a** Fractionated extracts from insect microbiomes are active in multiple murine models of drug-resistant infection. Less infective burden is seen in intraperitoneally treated mice after 8 h of infection. Each dot represents a unique fraction in one mouse study. (*n* = 15, 11, and 8 for *C. albicans*, *E. coli*, and *P. aeruginosa* models, respectively; center, median; box, upper and lower quantiles; whiskers, 1.5× interquartile range. **b** Most fractions from insect microbiomes show no hemolysis in cell-based assays. Safe indicates no toxicity at >100× concentration associated with efficacy. **c** The antifungal cyphomycin is produced by *Streptomyces* isolated from **d** the fungus-growing ant *Cyphomyrmex* sp. Photo credit: Alexander Wild **e** Cyphomycin-containing fractions show potency against the ant pathogen *Escovopsis* sp. (top left, bottom). **f** Purified cyphomycin exhibits potency against resistant pathogens. **g** Mouse candidiasis (*C. albicans*) models showcase reduced infection and a dose-like response to cyphomycin. Dots indicate individual mice

We next determined the genomic potential of insect-associated *Streptomyces* as a source of novel natural products. Core-genome phylogenetic studies of 120 strains (69 from insects) show that specific lineages of *Streptomyces* appear to be associated with insect hosts. Most insect-associated *Streptomyces* strains cluster together in discrete insect-associated lineages, often separated from soil *Streptomyces* lineages by millions of years^34^. This is further supported by our 16S rRNA gene phylogeny (Fig. 2). Thus, our findings suggest that *Streptomyces* from insects occupy unique evolutionary space for natural product discovery (Supplementary Figure 2A, subset shown in Fig. 4a). Through identification and characterization of the 4948 biosynthetic gene cluster (BGC) fragments present in these genomes via antiSMASH^35^, we show insect-associated *Streptomyces* harbor vast potential for natural product biosynthesis. BGCs were classified into 2672 families with BiG-SCAP^36^ over 71% of which were present within only a single genome (Supplementary Figure 2B, Supplementary Data 5). Among BGCs, 31% (1539) were flanked by at least 0.5 kb of sequence on either side, indicating the BGC was fully resolved. Notably, the distinct evolutionary lineages of *Streptomyces* enriched in insect-associated strains harbor much of the uncharacterized biosynthetic potential and have led to the discovery of new molecules (such as cyphomycin; see Fig. 5). Soil and insect *Streptomyces* had similar full-length BGC abundances in major biosynthetic cluster types and dedicated similar fractions of their genomes to secondary metabolism (11% and 12%, respectively; Supplementary Figure 2C-E). Uncharacterized BGCs are abundant across *Streptomyces*. Known BGCs are primarily found within well-studied, soil-derived species such as *S. griseus* and *S. coelicolor*, which may contribute to the issue of compound rediscovery during soil-centered sampling campaigns. The diversity of BGCs with respect to both sub-clade (Fig. 4a, phylogeny) and source indicates that the BGCs present within a *Streptomyces* genome are influenced by both phylogeny and ecology (Fig. 4b). Many BGC families had a Shannon entropy of zero with respect to their sub-clade, indicating their strong phylogenetic signal is likely related to a vertical evolutionary history. Other BGC families were source invariant, indicating that they are found exclusively within a specific ecological context (e.g., only within insect microbiomes). The influence of both phylogeny and ecology on BGC content further supports that *Streptomyces* from insect microbiomes represent a unique source of new antimicrobial chemistry.

### Ecology and phylogeny influence metabolomic profiles

We also compared the chemical fingerprints of insect-, plant-, and soil-sourced *Streptomyces* using untargeted liquid chromatography mass spectrometry (LC/MS) metabolomics of 120 strains (69 from insects, same strains as in genomics above). In accordance with genomic predictions of chemical diversity from BGCs, molecular features (MFs) from LC/MS support that metabolome chemical diversity is also heavily influenced by both phylogeny and ecology. Here, our detection of 7727 unique but reproducible (present in all three replicates, but in only 1 of the 120 strains examined) MFs, suggests substantial and specialized chemical diversity in our sampling. Over 4500 (59%) of MFs were found to be unique to a single strain and 80.2% were found in four or fewer strains (Supplementary Figure 2F, Supplementary Data 6). Most MFs (3244) are found solely in insect microbiomes within our sampling and 1179 and 2325 are present only in the Clade I and II evolutionary lineages^34^, respectively, further supporting the influence of both source and phylogeny on chemical diversity (Fig. 4c, d). Principal component analyses (PCA) of these metabolomes identified a *Streptomyces* sp. ISID311 as metabolic outlier within a group of related *Streptomyces* (Fig. 4e). A loadings analysis of these principal components identified MFs driving this strain’s uniqueness, including the new antifungal cyphomycin (Fig. 4f).

### Insect-*Streptomyces* fractions have potent in vivo activity

To validate that insect microbiomes represent a source of antimicrobials with activity against human pathogens, we conducted in vivo efficacy testing of chemical fractions obtained from different insect *Streptomyces* strains using murine models for *Pseudomonas aeruginosa*, *Escherichia coli*, and *Candida albicans* infections (*n* = 8, 11, and 15, respectively; Fig. 5a). Fractions with in vitro activity were subsequently analyzed using UPLC-MS. On average, fractions contained five or fewer compounds, which were compared to Antibase^37^, a database comprised mostly of microbial natural products. Fractions that contained *m/z* values indicating the presence of unknown/novel molecules and the absence of known molecules (within 5 parts per million) were used in subsequent in vivo mouse studies. Mice treated with fractions from insect-associated *Streptomyces* had a 1.32 and 0.77 log reduction in infectious burden of the Gram-negative pathogens *P. aeruginosa* or *E. coli*, respectively, compared to untreated controls. Mice infected with the fungal pathogen *C. albicans* had a 1.07 log reduction in infectious burden when treated with insect-*Streptomyces* fractions. Fractions from insect microbiome *Streptomyces* show no toxicity in hemolysis assays (concentrations of 50–500 µg mL^−1^) for 42 of 46 fractions (91%; Fig. 5b).

### Discovery of cyphomycin, a new antifungal compound

From one such fraction that was positive in mouse studies, we describe the new antifungal compound cyphomycin (Fig. 5c, Supplementary Figures 3 and 4) from a Brazilian *Streptomyces* (ISID311) isolated from the microbiome of the fungus-growing ant *Cyphomyrmex* sp. (Fig. 5d). The cyphomycin type 1 polyketide synthase BGC is 199 kb in length (Fig. 5c; polyketide open reading frames shown in blue, tailoring genes in yellow). It has biosynthetic similarity to the PM100117/8 family of antitumor compounds^38^ (BiG-SCAPE distance of 0.278) and structural similarity to the deplelides^39^, yet has key differences in the macrolide core and glycosylation (Supplementary Figures 3 and 4). The *Streptomyces* that produce PM100117/8 and deplelide have 99.04% and 98.6% 16S rRNA gene sequence similarity to ISID311, respectively. The PM100117/8 producing *Streptomyces* was isolated from a marine polychaete worm (genus *Filograna*) indicating potential for invertebrate-specialized structures and mechanisms in this class. Purified cyphomycin is active against multidrug-resistant fungal infections both in vitro and in vivo (Fig. 5e–g). Further, cyphomycin shows potent in vitro activity against both the ecologically relevant fungus-growing ant pathogen *Escovopsis* sp. (Fig. 5e) and the resistant human pathogens *Aspergillus fumigatus 11628* (triazole resistance), *C. glabrata 4720* (echinocandin resistance), and *C. auris B11211* (echinocandin, triazole, and amphotericin B resistance), with low minimum inhibitory concentrations (MICs) in vitro (Fig. 5f). Activity against fungi with resistance mechanisms to all three clinically used classes of antifungals indicates that further study exploring cyphomycin’s mechanism of action and resistance is warranted. In a neutropenic mouse disseminated candidiasis model^40^, infective burden was 5.38 ± 0.04 log10 CFU per kidney for fluconazole (4 mg kg^−1^), 5.2 ± 0.08 for amphotericin B (1 mg kg^−1^), 4.8 ± 0.03 for micafungin (4 mg kg^−1^), and 6.78 ± 0.02 for untreated controls (all *n* = 3). It is important to note that these doses produce the same plasma drug exposure seen in humans using standard dosing regimens^41^. In a single-dose study of cyphomycin in the same model, cyphomycin exhibited an in vivo dose–response with 0.56 and 0.66 log reduction of infectious burden compared to the start of therapy when treated with 20 and 40 mg kg^−1^ cyphomycin, respectively (Fig. 5g), demonstrating cyphomycin’s potential for treating clinically relevant pathogens in this industry-standard model of *Candida* infection^42^. 

## Discussion

Through our systematic assessment, we show insect microbiomes present a promising source of novel natural products. Our application of genomics, metabolomics, and ecologically optimized bioassays facilitates rapid screening of strains to explore their untapped chemical diversity. Although primarily thought of as soil microbes, we show *Streptomyces* from phylogenetically distinct lineages commonly form associations with diverse insect hosts. The unique chemical defenses of insect *Streptomyces*, such as mycangimycin^23^ and sceliphrolactam^27^, and other insect-associated Actinobacteria, such as dentigerumycin^19^ and selvamicin^21^, have been a fruitful discovery resource in the recent past. Inhibition of Gram-negative and fungal human pathogens (Fig. 3a–c) highlights the value of insect-associated *Streptomyces* as a source of bioactive molecules. Furthermore, these strains exhibit higher levels of inhibition than strains isolated from traditional sources (i.e., soil) that primarily result in the rediscovery of known compounds^43^. Genomic characterization of the biosynthetic potential of these strains and the metabolomic characterization of their produced metabolites highlight the unique chemical diversity of *Streptomyces* from insect microbiomes. Cyphomycin is an example of new chemistry from this innovative source. Importantly, the fractions discovered though in vitro and in silico screening retain high efficacy in mouse models of *C. albicans*, *E. coli*, and *P. aeruginosa* infections, and exhibit low toxicity in hemolysis assays (Fig. 5a, b). The unique ecological and evolutionary pressures on the chemical phenotypes of insect microbiomes may have further application in conservation, agriculture, and ecosystem health.

Insect microbiomes appear a particularly valuable source of antimicrobials for treating fungal diseases. High mortality rates and widespread emergence of resistance to many or all known antifungals have made invasive fungal infections a worldwide health burden^44^. The few effective antifungal therapeutics are often plagued with high toxicity and off-target effects and have done little to reduce the high mortality rates of multidrug-resistant fungal disease^44^. *Streptomyces* from insect microbiomes represent a prolific source of antifungal natural products and we show that insect strains exhibit significantly greater activity against fungi than soil *Streptomyces* (Fig. 3a). Further, we present that cyphomycin, a new molecule isolated from a fungus-growing ant microbiome, is active against multidrug resistant fungal pathogens and demonstrates in vivo efficacy in a commonly utilized infection model for PK/PD studies and FDA applications (Fig. 5f, g). Compared to existing FDA-approved antifungal agents, cyphomycin’s 0.25 µg mL^−1^ in vitro MIC against *C. albicans* K1 is similar to both amphotericin B and fluconazole (0.25 and 0.5 µg mL^−1^, respectively). It is important to note that the deplelides are not described as antifungals, but rather antitumor molecules. Further, we see no evidence of toxicity in mouse studies of cyphomycin and animals exhibited no observed physical or behavioral changes, suggesting that the insect-*Streptomyces* cyphomycin is a more specific molecule than the soil-derived deplelides that are generally toxic to eukaryotes. Together, activity against multidrug-resistant fungi in vitro coupled with high efficacy in in vivo mouse models of infection highlight cyphomycin’s potential as a drug lead to treat multidrug resistant fungal infections (Fig. 5f, g).

Antimicrobials developed from soil *Streptomyces* are the foundation of modern medicine and have saved countless lives. Widespread resistance to these compounds, in combination with the apparent exhaustion of soil *Streptomyces* as a source of new antimicrobials, represents an alarming health crisis—the rate of antimicrobial resistance continues to far outpace the discovery of novel antimicrobial natural products^2,14,45^. The promise of insect-associated *Streptomyces* as a new source of antimicrobials has the potential to reinvigorate the stagnated antibacterial and antifungal discovery pipelines. This source has potential far-reaching applications stemming from their ecological roles mediating pathogen dynamics associated with their insect hosts. Insects, through evolution, are predicted to have undergone millions of years of continual bioprospecting for active, defensive molecules; pathogen pressure selects for association with *Streptomyces* strains that produce efficacious antimicrobials. Furthermore, insect symbionts are uniquely suited to medicinal discovery, as their host associations appear to enrich for compounds with low toxicity to animals. Our validation of *Streptomyces* from insect microbiomes as a rich source of bioactive natural products demonstrates the extensive opportunities for antimicrobial discovery within the vast chemical diversity of these microbial communities.

## Methods

### Host collection

Host-associated strains were obtained from seven field collections (Florida, Hawaii, Alaska, New Mexico, Wisconsin, California, Brazil) from 2014 to 2016, with various Currie lab archival strains (Supplementary Data 1). Each host was collected using sterile forceps and deposited into a pre-sterilized, pre-barcoded container. Field collections focused heavily on insects that were not in direct contact with soil to avoid the possibility of soil contamination in subsequent surface isolation. Metadata recorded at each field site included: researcher information, date, location, GPS coordinates, micro and macro environment, host description, as well as photographs of each field-site. All specimens were assigned a unique host-identification number (HID) and stored at 4 °C.

### Processing and bacterial isolation

All hosts are photographed via dissecting scope and cataloged by HID.

Insect specimens were processed based on host integrity. If the sample was large enough and completely intact it was processed for external and internal microbial isolates. Large samples were also processed by particle method, using a sterilized surgical scalpel to remove portions of each specimen and placing each piece on an agar plate. If small, degraded or compromised, samples were processed using a combination method: the same procedure as the internal isolation without surface sterilization. External isolation involves transferring host specimen into a 1.5 mL microcentrifuge tube, adding 125*x (*x* = number of plates) µL phosphate-buffered saline (PBS), vortexing gently at 50% speed for 10 s, and transferring 100 µL to various media. To select for Actinobacteria, humic acid Agar (HV)^46^ and selective chitin media^47^ with 20 mL/1 L nystatin, and 10 mL/1 L cycloheximide added to select against fungal isolates were used. After plating for external isolates, a sterilization wash is preformed to isolate internal microbes. The same microcentrifuge tube from the external was filled with 1 mL of 70% ethanol and gently mixed by inversion for 1 min. Ethanol waste was removed and 1 mL of 1% bleach with 0.1% tween20 solution was added and mixed gently for 30 s by inversion. Supernatant was removed and host specimen was rinsed 3× using 1 mL PBS buffer for 10 s. After external sterilization, 125*x (*x* = number of plates) µL of PBS was added to the tube. The specimen was then ground-up using a sterile pestle inside the tube. Using a wide bore 200 µL tip, 100 µL of slurry was transferred onto a pre-labeled media plate (HV & chitin) and spread evenly. The last 100 µL was pipetted into a DNA voucher containing 900 µL of 95% ethanol for storage.

Plant tissue was vortexed in PBS and 100 µL plated. Remaining tissue was then surface sterilized, crushed, and plated similarly. Particle plates were generated for some plants, where we plant tissue was placed directly on plates. The same selective protocols as above were used.

Soil was treated similarly by re-suspending 1 g into 1 mL of PBS, followed by a 1e−5 serial dilution on chitin and HV. Colonies were then picked.

For all sources, HV and chitin isolation plates were incubated aerobically at room temperature (28 °C) and checked for bacterial growth at 14 d, 30 d, and 90 d. Colonies exhibiting characteristic Actinobacterial morphology^48^ were assigned a strain identification number before isolation onto fresh HV or chitin media and incubation at 28 °C.

### 16S sequencing and phylogenetic placement

Genomic DNA was extracted from isolates using the Powersoil DNA isolation kit according to manufacturer’s specifications (MoBio). Polymerase chain reaction (PCR) was performed using the universal bacterial primer set 27 F (5′-AGA GTT TGA TCM TGG CTC AG-3′) and 1496 R (5′-CGG TTA CCT TGT TAC GAC TT-3′), of hypervariable regions V1-V9. PCR was performed in a standard 25 µL reaction with 1 µL DNA template (20 ng µL^−1^), 12.5 µL EconoTaq (Lucigen corporation), 1 µL combined 10 µM primers, and 12.5 µL water. Initial denaturation for 3 min at 95 °C was followed by 3 min annealing at 58 °C followed by 35 cycles of 10 s at 96 °C and 2 min at 72 °C, and a post-cycle extension at 72 °C for 7 min. Amplicons were confirmed on 1.2% agarose electrophoresis gel prior to cleanup wth Wizard SV Gel and PCR system (Promega). Big Dye sequencing reaction was then performed followed by a secondary clean-up prior to submission to UW Biotech for analysis (University of Wisconsin-Madison). 16S sequences were searched with blastn against a database of representative genomes from each clade in the *Streptomyces* phylogeny (see Genome sequencing and Core-genome phylogeny below). Hits with the best bitscore were assigned to that clade. Within clade hits 16S were then aligned with MAFFT v7.245^49^ and treed with FastTree2^50^ under the GTR substitution model.

### Inhibition bioassays and scoring

Isolates were inoculated along the center of wells. Each well contained 3 mL of yeast peptone mannitol (YPM) agar (2 g yeast extract, 2 g peptone, 4 g mannitol, 15 g agar, 1 L H_2_O). Actinobacteria were incubated at 28 °C for 5 d prior to the addition of the test pathogens. For fungal pathogens, spore stocks of each fungal strain were diluted 1:10 (Supplementary Data 3). For bacterial and yeast pathogens 3 mL of broth containing the microorganism was inoculated into sterile 14 mL tubes with Luria-Bertani (LB) or yeast peptone dextrose (YPD), respectively. Cultures were shaken overnight at 28 °C and diluted 1:10 (Supplementary Data 3). Diluted cultures were used to inoculate 3 µL in the center of the well and the non-pathogen controls. Plates were maintained at 28 °C for 7 d. Inhibition was scored in binary, as 0 (no inhibition) or 1 (inhibition). For a subset of isolates, experimental wells were assigned a rating from 0 to 3 depending on the level of inhibition (0—no inhibition, 1—slight inhibition, 2—presence of a zone of inhibition, 3—complete inhibition).

Inhibition fraction was determined by taking the average inhibition of Strain *X* vs. Pathogen *Y* to account for variance if multiple *X* by *Y* challenges were run. This was done for all pathogens against *X*. Gram-positive, gram-negative, and fungal averages were then computed to find the inhibition fraction for *X*. Strains were grouped by source and *p*-values were calculated by *T*-test with Benjamini/Yekutieli correction^51^. Fuzzy clustering was used to group inhibitory profiles using Mfuzz^52^.

### Genome sequencing and assembly

120 strains were selected for whole genome sequencing based on 16S rRNA gene sequence so to span the *Streptomyces* phylogeny, including outgroups. Cultures were grown in rich medium supplemented with 0.5% glycine and cells were harvested by centrifugation. Cells were washed with 10.3% sucrose, resuspended in lysozyme solution (3 mg mL^−1^ lysozyme, Sigma, in 0.3 M sucrose, 25 mM Tris pH 8, 25 mM EDTA pH 8), and incubated at 37 °C for 30 min. Proteinase K (Thermo Fisher; 20 mg mL^−1^) was added before incubation for 15 min at 42 °C. Cells were lysed by adding 2% SDS and rocking for 5 min until lysis was complete. Neutral phenol and chloroform were added, and tubes were gently shaken until uniformly white. After centrifugation, the top layer was transferred to 3 M sodium acetate pH 6 and isopropanol. Tubes were gently mixed until DNA appeared. DNA was pelleted, supernatant was removed, and the pellet was resuspended in TE with 0.2 mg mL^−1^ RNaseA. Tubes were incubated 15 min at 28 °C before adding 5 M NaCl and CTAB/NaCl solution. Tubes were incubated for 10 min at 55 °C and cooled to 28 °C. CHCl_3_ was added, tubes were gently shaken, and spun for 10 min at 28 °C. The top layer was transferred to a new tube and extracted again with phenol and chloroform, followed by extraction with chloroform and precipitation with 3 M sodium acetate pH 6 and isopropanol. The pellet was washed in 70% ethanol and resuspended in water. DNA was quantified, checked for purity, and run on a gel to verify high molecular weight. Genomic DNA libraries for Illumina MiSeq 2 × 300 bp paired-end sequencing were prepared by the University of Wisconsin-Madison Biotechnology Center (TruSeq). Reads were corrected with MUSKET v1.1^53^, paired-ends were merged with FLASH v1.2.7^54^, and assembled with SPAdes v3.11.0^55^.

### Core-genome phylogeny

A genome-based, multilocus phylogeny was generated using 93 TIGRFAM proteins in the core bacterial protein set (GenProp0799; http://www.jcvi.org/cgi-bin/genome-properties/GenomePropDefinition.cgi?prop_acc=GenProp0799). Genes were called with prodigal v2.6.0^56^ and GenProp0799 profile Hidden Markov Models were used to search each genome. HMMER v3.1b2^57^ was used to identify protein sequences for each protein family. Each family was then aligned using MAFFT v7.245^49^. Alignments were then converted to codon alignments and concatenated. The multi-locus phylogeny was generated using RAxML v8.1.24^58^ under the GTRgamma substitution model with 100 rapid bootstraps.

### Analysis of biosynthetic gene clusters

BGCs were identified within each genome with antiSMASH v4.0^35^. BGCs were determined to be full-length if 0.5 kb of flanking sequence existed between cluster boundaries and the end of the contig. BGCs were used to identify BGC groups via BiG-SCAPE (https://git.wageningenur.nl/medema-group/BiG-SCAPE/) under --hybrid and --mode glocal settings at a distance cutoff of 0.5. BGCs from our dataset were combined with all available BGCs from MIBiG v1.3^59^ and again groups were called in BiG-SCAPE. Distances <0.5 were called similar, between 0.5 and 0.75 divergent, and over 0.75 uncharacterized. Shannon entropy with respect to phylogeny, strain source (e.g., insect-associated, soil, plant), and insect-host order were calculated for BGC groups.

### Untargeted LC/MS

*Streptomyces* were cultured on YPM agar media and grown for up to 14 days or until sporulation. Two 8 mm diameter cores were sampled directly and extracted with 2 mL of MeOH for 30 min. Extracts were transferred to new vials and vacuum dried. Extracts were dissolved in 100 μL of MeOH, followed by 1 mL of Milli-Q water. Solid phase extraction (SPE) was conducted using Biotage: EVOLUTE ABN (25 mg, 1 mL). Samples were loaded following conditioning with MeOH and then Milli-Q water. Extracts were washed with Milli-Q water to remove media components and primary metabolites and then eluted with MeOH into LC/MS certified vials. Mass spectra were collected using a Bruker MaXis ESI-Q-TOF mass spectrometer. Liquid chromatography was conducted using a Waters Acquity UPLC on a RP-C18 column (Phenomenex Kinetex 2.6 μm, 2.1 mm × 100 mm). Both instruments were operated using Bruker Hystar software. A linear MeOH/H_2_O (0.1% formic acid) gradient was used beginning at 10%/90% and reaching 97%/3% in 12 min and held for 3.5 min. The column was set to initial conditions in 0.5 min and re-equilibrated for 3.5 min before subsequent runs. The flow rate was 0.3 mL min^−1^. Full-scan mass spectra (*m*/*z* 150–1550) was collected in positive ESI mode. The following parameters were used: capillary, 4500 V; nebulizer, 1.2 bar; dry gas flow rate, 8.0 L min^−1^; dry gas temperature, 205 °C; scan rate, 2 Hz. Automatic internal calibration was conducted after each run by introducing Tune Mix (Agilent, ESI-L low concentration) through a divert valve during re-equilibration. Bucket tables from LC/MS were generated in Bruker Profile Analysis 2.1 with Molecular features identified as follows: S/N threshold, 5; correlation coefficient threshold, 0.7; minimum compound length, 10 spectra; smoothing width, 1; bucketing basis, M + H. Buckets were generated from LC/MS traces between 120 and 840 s and for *m*/*z* ratios between 150 and 1500. Advanced bucketing was used with ΔRT = 20 s and Δ*m*/*z* = 20 mDa. Buckets were normalized to the sum of buckets values in the analysis. Mass to charge ratios were manually cross-referenced with Antibase^37^ within 5 parts per million of M, M + H, and M + Na adduct states.

### Hemolysis assay

Assays were performed in 384-well plates using sheep blood (0.1% triton as the positive control). Sheep’s blood (Ward’s Science) was washed with PBS and diluted to a concentration of 6 × 10^7^ red blood cells per mL. A volume of 50 µL of blood was incubated with compound for 1 h, and subsequently pelleted at 4000 rpm for 10 min. Thirty microliters of supernatant were transferred to a clear plate and OD570 was read. An increase in OD indicated the red blood cell lysis and hemolytic activity.

### Mouse studies

All mouse experiments and protocols received ethical approval from the University of Wisconsin Institutional Animal Care and Use Committee.

### Candida model

Six-week-old, specific-pathogen-free, female ICR/Swiss mice weighing 23–27 g were used for all studies (Harlan Sprague-Dawley, Indianapolis, IN). Animals were maintained in accordance with the criteria of the Association for Assessment and Accreditation of Laboratory Animal Care. All animal studies were approved by the Animal Research Committee of the William S. Middleton Memorial Veterans Hospital. Mice were rendered neutropenic (neutrophils, 100 per mm^3^) by injection with cyclophosphamide (Mead Johnson Pharmaceuticals, Evansville, IN) subcutaneously 4 days (150 mg kg^−1^) and 1 day (100 mg kg^−1^) before infection and 2 days after infection (100 mg kg^−1^). Previous studies have shown neutropenia (neutrophils, 100 per mm^3^) in this model for the 96-h study period^42^. Organisms were subcultured on SDA 24 h prior to infection. Inoculum was prepared by placing three to five colonies into 5 mL of sterile pyrogen-free 0.9% saline warmed to 35 °C. The final inoculum was adjusted to a 0.6 transmittance at 530 nm. Fungal counts of the inoculum determined by viable counts of *C. albicans* on SDA were 6.29 ± 0.03, 6.15 ± 0.10, and 6.30 ± 0.07 log10 CFU mL^−1^, respectively. Disseminated infection with the Candida was achieved by injection of 0.1 mL of inoculum via the lateral tail vein 2 h prior to the start of drug therapy. Treatment period was 8 h. Animals were sacrificed by CO_2_ asphyxiation. Kidneys of each mouse were removed and placed in sterile 0.9% saline at 4 °C. The homogenate was serially diluted and aliquots were plated on SDA for viable fungal colony counts after incubation for 24 h at 35 °C. The lower limit of detection was 100 CFU mL^−1^. Results were expressed as the mean number of CFU per kidney for three mice. No-treatment and zero-hour controls were included in all experiments.

### Mouse bacterial thigh infection model

Animals for the present studies were maintained in accordance with the criteria of the Association for Assessment and Accreditation of Laboratory Animal Care. All animal studies were approved by the Animal Research Committee of the William S. Middleton Memorial VA Hospital.

Six-week-old, specific-pathogen-free, female ICR/Swiss mice weighing 23–27 g were used for all studies (Harlan Sprague-Dawley, Indianapolis, IN). Mice were rendered neutropenic (neutrophil count, <100 mm^−3^) by injecting them with cyclophosphamide (Mead Johnson Pharmaceuticals, Evansville, IN) subcutaneously 4 days (150 mg kg^−1^) and 1 day (100 mg kg^−1^) before thigh infection. Previous studies have shown that this regimen produces neutropenia in this model for 5 days^40^. Broth cultures of freshly plated bacteria were grown overnight to logarithmic phase to an absorbance at 580 nm of 0.3 (Spectronic 88; Bausch and Lomb, Rochester, NY). After 1:10 dilution into fresh Mueller–Hinton broth, the bacterial counts of the inoculum ranged from 10^7.0^ to 10^7.4^ CFU mL^−1^. Thigh infections with each of the isolates were produced by injection of 0.1 mL of inoculum into the thighs of isoflurane-anesthetized mice. Antibacterial therapy was initiated 2 h after the infection procedure. After 8 h, the animals were euthanized, and the thighs were aseptically removed, homogenized, and plated for determination of the number of CFU. No-treatment controls were included in all experiments.

### In vivo murine studies

To analyze fractions as candidates for in vivo study, we used Core-shell technology UPLC columns which provide extensive sensitivity and dynamic range for small molecules^60^ (Fig. 5a). The threshold in terms of accuracy was set at 3–5 ppm, well beyond the RMSD accuracy of a Bruker MaXis 4G (specifications of better than 1 PPM). Additionally, NMR data for each fraction were acquired using a 1.7 mm cryoprobe, the most sensitive NMR system for 1 H analysis. Prior to in vivo testing, samples were analyzed by NMR and LCMS to ensure characteristics of each sample matched to the original sample that exhibited in vitro activity. Since ELSD data were used to quantify each fraction, we were able to account for the mass of fraction during preparation of the LCMS sample. Acquisitions were performed such that ion counts of 10^6^ or greater were observed for the highest abundance ions, which facilitated detection of minor components. While there is a possibility of a known compound that does not ionize by ESI, studies indicate that 93% of microbial natural products ionize by positive ESI^61^.

LCT-ToF was used (as in Nielsen et al.^61^ where 93% ionization of microbial natural products was reported). In addition, a Bruker MaXis 4G with an Acquity UPLC front end and core shell columns was used to maximize sensitivity for ESI-MS of small molecules (dynamic range of 4.7 orders of magnitude). As such, ion counts in the millions maintain accuracy and appropriate peak shape. Low abundance ions (below 100 counts) can also be detected (noise is ~30–50 counts). Thus, with the increased accuracy and range of our instrument, the 93% estimate of microbial natural products from Nielsen et al. is a lower bound. Some lipids (e.g., those without carboxylic acid groups) and terpenes (e.g., those with few oxygens) ionize poorly and may be missed by ESI-MS, but within actinomycetes, we encounter few terpenes being produced under laboratory conditions.

Mice were treated 2 h after infection and sampled 6–8 h after treatment.

Bacteria were grown in three different media (A media, ISP-2, YPM, and A-Medium; 100 mL each) with Diaion HP20 (7% by weight) and shaken at 200 rpm at 28 °C for 7 days. Filtered HP20 was washed with water and extracted with acetone. The acetone extracts from the three media were combined and followed by solid phase extraction using ENV^+^ columns to generate four fractions. These four fractions were further purified by HPLC to generate 80 fractions for each strain in a 96-well plate. High-throughput screening was used to evaluate the antimicrobial activity of these fractions. Dereplication based on 1.7 mm NMR and HRMS was used to select promising hits. Select strains were re-grown in the large scale in the three media, and followed the same processor mentioned before to generate the fractions containing potential novel active compounds. These fractions were formulated and tested in vivo activity (Fig. 5a).

### In vitro MIC susceptibility testing

MICs of cyphomycin for the *Candida* isolates were determined using a broth microdilution method in accordance with the guidelines presented in Clinical and Laboratory Standards Institute (CLSI) document M27-A3 for *Candida* and M38-A2 for *Aspergillus*^40,42^. MIC values of cyphomycin were defined as the lowest concentration at which a prominent decrease in growth turbidity (i.e., 50% reduction in growth determined spectrophotometrically) relative to the turbidity of the compound-free control at 600 nm was observed. Median MIC from replicate assays is reported.

### Cyphomycin—culturing and isolation

*Streptomyces* ISID311 was grown in A-medium broth (20 g soluble starch, 10 g glucose, 5 g peptone, 5 g yeast extract, 5 g CaCO_3_ per liter) using Fernbach flasks [5× (1 L of medium in 2.8 L + 70 g of HP20)] and shaken for 7 days at 28 °C and 200 rpm. The HP20 was filtered and washed with distillated water and soaked with acetone. Organic solvent was filtered, vacuum dried, and partitioned with ethyl acetate/water. Organic phase was separated and dried to give the crude extract (2.3044 g). Extract was purified by SPE-C18 (55 µm, 20 g) using the following gradient: 200 mL (20% MeOH-H_2_O, **A1:** 72.8 mg); 200 mL (40% MeOH-H_2_O, **A2:** 152.5 mg); 200 mL (60% MeOH-H_2_O, **A3:** 132.3 mg); and 200 mL (100% MeOH, **A4:** 1.9349 g).

**A4** was purified by semi-preparative HPLC using C_18_ semipreparative column (Phenomenex Luna, C_18_(2), 5 µm, 250 × 10 mm) and the following gradient of MeOH and H_2_O (containing 0.1% of acetic acid) at 4 mL min^−1^: min 1–20 (linear gradient from 80% MeOH-H_2_O to 100% MeOH); min 20–22 (isocratic flow of 100% MeOH); min 22–22.5 (linear gradient from 100% MeOH to 80% MeOH-H_2_O); and min 22.5–27.5 (isocratic flow of 80% MeOH-H_2_O) to yield cyphomycin (89.0 mg, *t*_R_ = 11.4 min).

### Cyphomycin—structural determination

The molecular formula of cyphomycin was C_77_H_122_O_26_ based on positive ion HRESIMS ([M + H]^+^ *m/z* 1463.8282, err 1.0 ppm). The ^1^H and ^13^C NMR spectral data of **2** are shown in Supplementary Figure 3AI. The ^13^C NMR spectrum showed 77 signals assigned to 12 methyl, 18 methylene, 36 methine, 5 carbonyl carbons, 4 tertiary carbons sp^2^ and 2 quaternary carbon groups by multiplicity-edited *g*HSQC experiment. The COSY and HSQC-TOCSY spectra revealed connectivity from H-2 to H-15 and H-18 to H-43 (Supplementary Figure 3AF).

Connection of these two spin systems was revealed with the HMBC correlation of H-18 (δ_H_ 2.09 and 1.33) to C-16 and C-17; H-15 (δ_H_ 4.69) to C-16; and H-35 (δ_H_ 5.14) to C-1. Geometry of the two double bonds of macrolide were determine to be *E* configured by coupling constants of ^3^*J*_H-2,H-3_ (15.8 Hz) and ^3^*J*_H-32,H-33_ (15.6 Hz). The positions of methine groups H-4, H-14, H-34, H-36, H-38 and H-40 were assigned by ^1^H-^1^H COSY correlations to methyl groups H-44, H-45, H-46, H-47, H-48 and H-49, respectively. The methyl group H-43 was assigned as terminal group of side chain by its triplet multiplicity in ^1^H NMR spectrum and HMBC correlations to C-41 and C-42. Positions of oxygenated methine and methylene groups in macrolactone moiety were established by HMBC, COSY and HSQC-TOCSY correlations, and comparison of NMR data of compounds PM100117/8^38^. Difference in macrolactone moiety of compound **1** versus PM100117/8 is the additional methyl group H-44 attached to C-4. Other similar macrolactones of 36 members are described in GT-35 and Deplelides A and B^39^.

The presence of two sugar units was evidenced by signal of ^1^H and ^13^C NMR of two anomeric protons and carbons at δ_H_ 5.01 (H-1´) and δ_H_ 4.58 (H-1´´); and δ_C_ 96.4 (C-1´) and δ_C_ 104.0 (C-1´´), respectively. The first was established as α-axenose by HMBC, COSY, ROE correlations and NMR data comparison with literature^38^ (Supplementary Figure 3AG).

There are two ^1^H spin systems from H-1´ to H-2´ and from H-4´ to H-6´; and HMBC correlations from methyl H-7´ to C-2´, C-3´ and C4´. Connection of this sugar to side chain of macrolactone was observed with HMBC correlations of H-1´ to C-41. Small coupling constant of H-1´ and H-4´suggested they should be in equatorial orientation. ROE correlation of H-41 to H-5´ supported axial orientation of H-5´.

The second sugar was established as *β*-amicetose. It was constructed by the ^1^H spin system from H-1´´ to H-6´´. Large coupling constants ^3^*J*_H-1´´,H-2´´ax_ (7.6 Hz) and ^3^*J*_H-4´´,H-5´´_ (9.3 Hz); and ROE correlation between H-1´´ and H-5´´ suggested axial orientation of H-1´´, H-4´´ and H-5´´. Connection of both sugars was observed by the HMBC correlation of H-1´´ to C-4´. The HMBC correlation of H-4´´ to C-50 connected the *β*-amicetose with the ester carbonyl group of naphthoquinone derivative moiety. ^1^H NMR signals for H-54 (δ_H_ 8.01), H-61 (δ_H_ 8.07) and H-62 (δ_H_ 7.79); ^1^H-^1^H coupling constants ^3^*J*_H-54,H-62_ (1.3 Hz) and ^3^*J*_H-61,H-62_ (8.0 Hz); and ^1^H-^1^H COSY correlations between H-54/H-62 and H-61/H-62 indicated the presence of an aromatic ring trisubstituted moiety. HMBC correlations of H-54 to C-56; H-61 to C-55 and C-59; H-62 to C-60; H-57 to C-55, C-59 and C-64; and H-64 to C-57, C-58, and C-59 indicated the presence of a quinone moiety methylated in C-58 and connected to the aromatic ring. HMBC correlation of H-63 to C-50, C-51 and C-52; H-52 to C-50 and C-53; H-54 and H-62 to C-52; evidence the connection of C-50, C-51, C-52, C-53 and C-63 to aromatic ring of naphthoquinone moiety. Compound **2** was named cyphomycin.

## Supplementary information

Supplementary information

Description of Additional Supplementary Files

Supplementary Data 1

Supplementary Data 2

Supplementary Data 3

Supplementary Data 4

Supplementary Data 5

Supplementary Data 6

## Data Availability

Genomic data can be found at DOI: 10.5281/zenodo.2436565. All other data are available in the main text or the supplementary materials; Permits for collections and accessing genetic resources in Brazil were issued by SISBIO #46555–5 and CNPq #010936/2014–9. Costa Rican collecting permits were issued by the Comisión Institucional de Biodiversidad (Institutional Biodiversity Committee, University of Costa Rica; Resolutions # 012 and 020; Material Transfer Agreement MTA VI-4307–2013) and authorized by La Selva Biological Station and Las Brisas Nature Reserve. A modified version of the southern pine beetle (Fig. 1) photo from Erich G. Vallery is used under the Creative Commons Attribution 3.0 License. Photos of *Cyphomyrmex* (Figs. 1 and 5) are used under a perpetual commercial license from Alexander Wild.
