# Supplementary material for: The antimicrobial potential of Streptomyces from insect microbiomes
Source: Nat Commun. 2019 Jan 31;10:516. doi: 10.1038/s41467-019-08438-0 (PMC6355912; doi:10.1038/s41467-019-08438-0)
Supplement: Supplementary file 1 — Supplementary information [file 41467_2019_8438_MOESM1_ESM.pdf]

Supplementary information

Chevrette et al.

The antimicrobial potential of *Streptomyces* from insect microbiomes

Supplementary Figure 1: Inhibition bioassays.

Supplementary Figure 2: Genomics and metabolomics.

Supplementary Figure 3: Structural determination of cyphomycin.

Supplementary Figure 4: Proposed biosynthetic logic of cyphomycin.

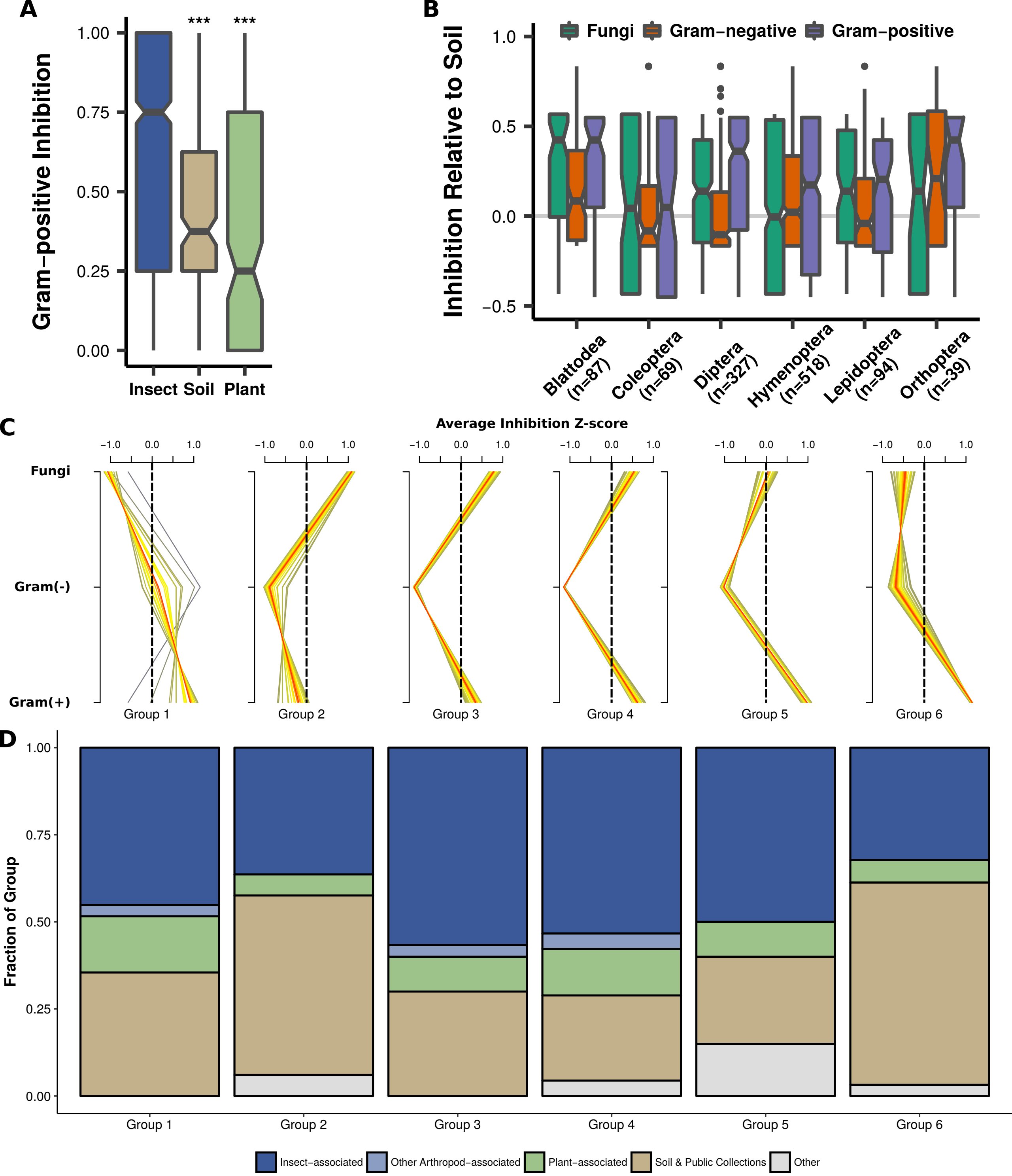

Supplementary Figure 1. Inhibition bioassays. (A) Gram-positive inhibition by *Streptomyces* strains from insect, soil, or plant sources. Insect microbiome *Streptomyces* show significantly higher inhibition against Gram-positive bacteria than do soil- or plant-sourced *Streptomyces*. (n=1162, 186, and 178 for insect, soil, and plant, respectively; ***p<1e-3; t-test, BY correction) (B) *Streptomyces* from insect hosts of different orders have differential activity in inhibition bioassays against Gram-positive bacteria, Gram-negative bacteria, and fungi. On average, *Streptomyces* from these hosts show higher activity compared to soil *Streptomyces*. (n=87, 69, 327, 518, 94, and 39 for Blattodea, Coleoptera, Diptera, Hymenoptera, Lepidoptera, and Orthoptera, respectively). A, B: center, median; box, upper and lower quantiles; notches, 95% confidence; whiskers, 1.5x interquartile range; points, outliers. (C) Fuzzy clustering of *Streptomyces* isolates identified groups of strains by their patterns of inhibitory activity against Gram-positive bacteria, Gram-negative bacteria, and fungi. Z-scores of inhibitory activities (deviance from the mean, 0) are shown for fungi, Gram-negative bacteria, and Gram-positive bacteria. Each line corresponds to an isolate within a given group. The color of the line denotes how strongly a strain fits into the group. For example, group 1 shows much less than average fungal activity but relatively high Gram-positive activity. Some strains within this group have high and relatively specific Gram-negative activity. In group 2, there is average Gram-positive activity, low Gram-negative activity, and high fungal activity. (D) Membership of these groups shows patterns of inhibition correspond to patterns of community membership. For example, group 6 has a higher proportion of soil strains than does group 3.

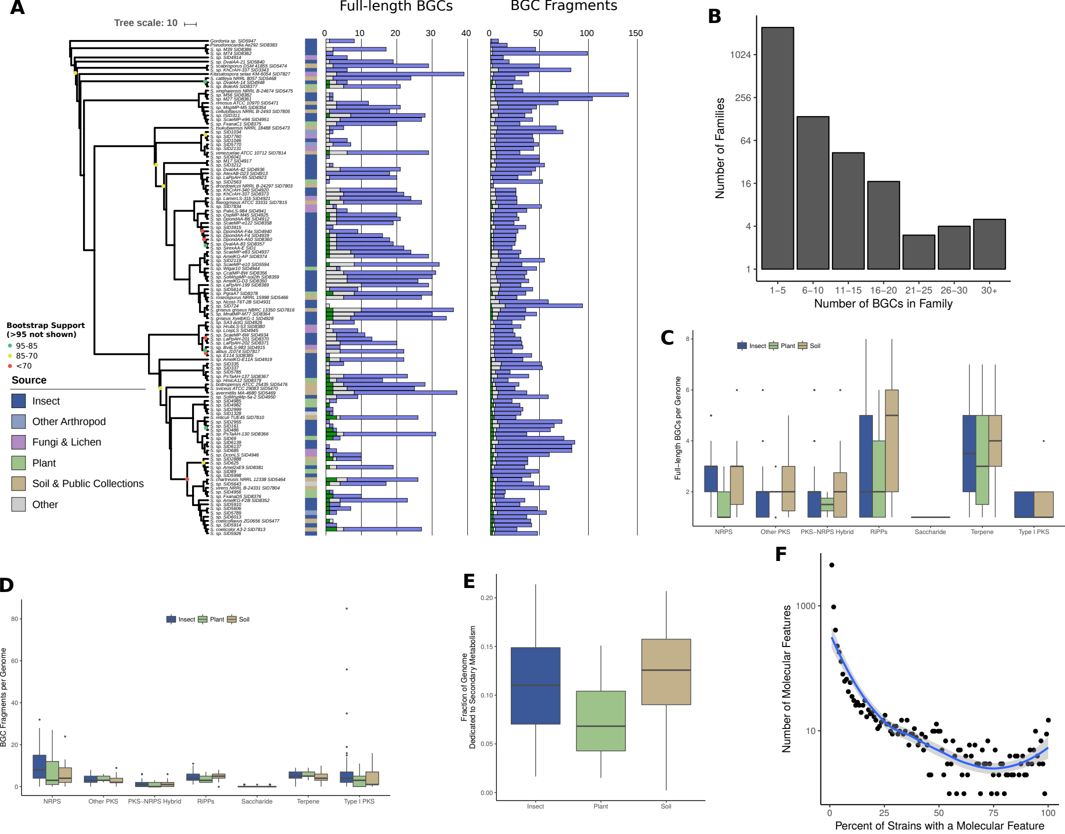

Supplementary Figure 2. Genomics and metabolomics. (A) Core-genome phylogeny based on a concatenated 93-gene multilocus sequence of core, single copy bacterial genes (JCVI GenProp0799; see http://www.jcvi.org/cgi-bin/genome-properties/GenomePropDefinition.cgi?prop_acc=GenProp0799). *Gordonia* sp. SID5947 was used as an outgroup. Nodes with bootstrap support below 95 are marked. Source (e.g. insect-associated, other arthropod-associated, fungi-associated, plant-associated, or soil) is shown in the color strip to the right of the tree. Full-length BGCs (BGCs with at least 0.5kb of DNA on either side of a cluster boundary) and BGC fragments for each genome are shown at right. All were compared to the known BGCs in the MIBiG database. As in Figure 2A, green denotes a BiG-SCAPE distance of less than 0.5 (similar), grey is a distance between 0.5 and 0.75 (divergent), and blue is a distance over 0.75 (uncharacterized). (B) The distribution of the number of BGCs in each family, as defined by a BiG-SCAPE distance of 0.5 or less, in *Streptomyces*. Most BGC families have 5 or less examples in our dataset. (C) The number of BGC fragments per genome by type for each of insect-, plant-, or soil-sourced samples. (D) The number of full-length BGCs per genome by type for each of insect-, plant-, or soil-sourced samples. (E) The fraction of the genome dedicated to secondary metabolism for insect, plant, or soil isolates. C, D, E: n=69, 21, and 13 for insect, soil, and plant respectively; center, median; box, upper and lower quantiles; whiskers, 1.5x interquartile range; points, outliers. (F) Distribution of LC/MS molecular features across Streptomyces. As with BGCs (panel B above) most molecular features are in a small percentage of strains within the dataset. A LOESS regression curve is shown in blue with 95% confidence intervals shown in grey.

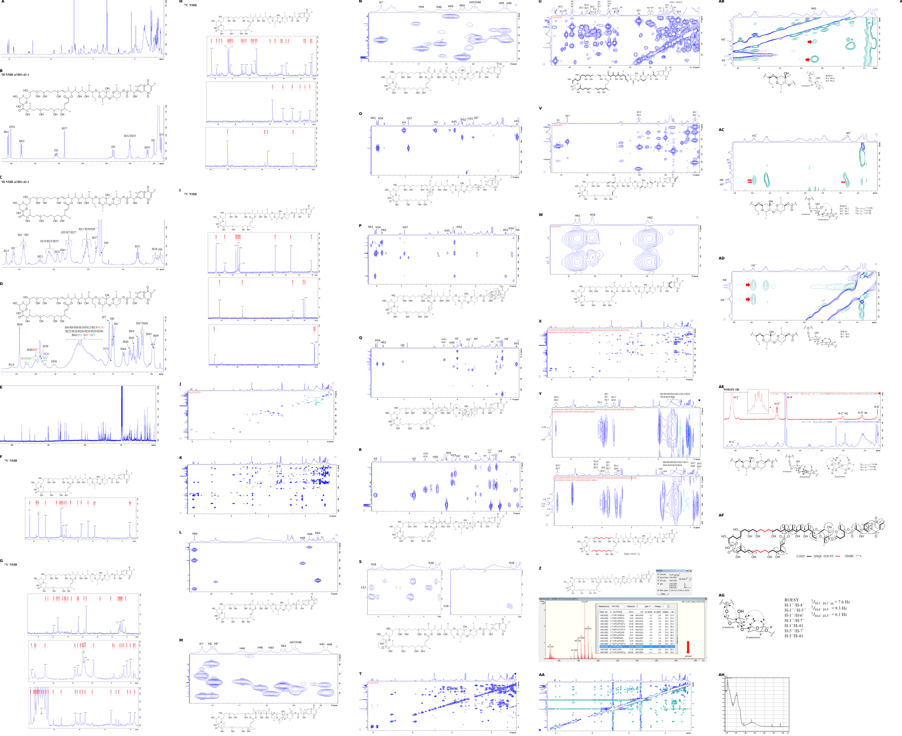

AI

No.	δ_C	δ_H, mult. (J in Hz)	No.	δ_C	δ_H, mult. (J in Hz)
**1**	168.7	---	**40**	38.5	2.00, m
**2**	122.1	5.86, br d (15.8)	**41**	80.5	3.88, m
**3**	153.5	7.06, dd (15.8, 6.8)	**42**	22.9	1.64, m
					1.36, m
**4**	43.5	2.47, m	**43**	11.5	0.98, t (7.2)
**5**	74.2	3.78, m	**44**	14.1	1.09, d (6.7)
**6**	41.7	1.60, m	**45**	13.6	0.79, d (6.4)
		1.54, m			
**7**	71.8	3.78, m	**46**	17.3	1.03, d (6.5)
**8**	38.9	1.32-1.62, m	**47**	9.7	0.90, d (6.8)
**9**	23.0	1.45-1.68, m	**48**	5.0	0.90, d (6.8)
**10**	38.5	1.32-1.62, m	**49**	10.6	0.78, d (6.4)
**11**	72.9	3.51, m	**1′**	96.4	5.01, br s
**12**	36.4	1.38-1.47, m	**2′**	37.9	1.93, dd (3.70, 14.0)
		1.53-1.63 m			1.55, d (14.0)
**13**	31.9	1.69, m	**3′**	71.2	---
		1.30, m			
**14**	36.2	2.25, m	**4′**	81.7	3.31, m
**15**	75.8	4.69, d (2.2)	**5′**	64.7	4.44, m
**16**	210.6	---	**6′**	17.3	1.21, d (6.6)
**17**	99.4	---	**7′**	27.5	1.24, s
**18**	41.1	2.09, m	**1′′**	104.0	4.58, br d (7.6)
		1.33, m			
**19**	64.9	4.08, m	**2′′**	31.3	2.01, m
					1.56, m
**20**	42.0	1.96, m	**3′′**	28.6	2.10, m
		1.21, m			1.56, m
**21**	67.7	4.20, m	**4′′**	74.4	4.45, m
**22**	45.6	1.45-1.74, m	**5′′**	74.5	3.54, dq (6.1, 9.3)
**23**	65.4	4.08, m	**6′′**	18.5	1.19, d (6.1)
**24**	46.3	1.43-1.67, m	**50**	175.5	---
**25**	68.2	4.04, m	**51**	48.9	2.78, dq (8.5, 7.1)
**26**	46.0	1.40-1.76, m	**52**	76.4	4.86, d (8.5)
**27**	71.3	3.79, m	**53**	150.3	---
**28**	38.4	1.32-1.62, m	**54**	125.3	8.01, br s
**29**	22.7	1.51, m	**55**	133.5	---
**30**	38.4	1.32-1.62, m	**56**	186.2	---
**31**	72.4	3.94, m	**57**	136.5	6.88, br d (1.4)
**32**	135.2	5.53, dd (15.6, 4.5)	**58**	149.8	---
**33**	133.1	5.49, dd (15.6, 7.2)	**59**	186.3	---
**34**	40.4	2.54, m	**60**	132.9	---
**35**	77.3	5.14, br d (9.5)	**61**	127.6	8.07, d (8.0)
**36**	38.7	1.97, m	**62**	133.2	7.79, dd (8.0, 1.3)
**37**	78.5	3.39, br d (9.5)	**63**	14.3	0.94, d (7.1)
**38**	36.0	1.81, m	**64**	16.4	2.17, s
**39**	79.4	3.52, m			

Supplementary Figure 3. Structural determination of cyphomycin. (A) 1H NMR (CD3OD, 500 MHz). (B) 1H NMR (CD3OD, 500 MHz). Expansion 1. (C) 1H NMR (CD3OD, 500 MHz). Expansion 2. (D) 1H NMR (CD3OD, 500 MHz). Expansion 3. (E) 13C NMR (CD3OD, 125 MHz). (F) 13C NMR (CD3OD, 125 MHz). Expansion 1. (G) 13C NMR (CD3OD, 125 MHz). Expansion 2. (H) 13C NMR (CD3OD, 125 MHz). Expansion 3. (I) 13C NMR (CD3OD, 125 MHz). Expansion 4. (J) Multiplicity-edited gHSQC (CD3OD, 500 MHz). (K) gHMBC (CD3OD, 500 MHz). (L) gHMBC (CD3OD, 500 MHz). Expansion 1. (M) gHMBC (CD3OD, 500 MHz). Expansion 2. (N) gHMBC (CD3OD, 500 MHz). Expansion 3. (O) gHMBC (CD3OD, 500 MHz). Expansion 4. (P) gHMBC (CD3OD, 500 MHz). Expansion 5. (Q) gHMBC (CD3OD, 500 MHz). Expansion 6. (R) gHMBC (CD3OD, 500 MHz). Expansion 7. (S) gHMBC (CD3OD, 500 MHz). Expansion 8. (T) gCOSY (CD3OD, 500 MHz). (U) gCOSY (CD3OD, 500 MHz). Expansion 1. (V) gCOSY (CD3OD, 500 MHz). Expansion 2. (W) gCOSY (CD3OD, 500 MHz). Expansion 3. (X) gHSQC-TOCSY (CD3OD, 500 MHz). (Y) gHSQC-TOCSY (CD3OD, 500 MHz). Expansion 1. (Z) HRESMS spectrum. (AA) gROESY (CD3OD, 500 MHz). (AB) gROESY (CD3OD, 500 MHz). Expansion 1. (AC) gROESY (CD3OD, 500 MHz). Expansion 2. (AD) gROESY (CD3OD, 500 MHz). Expansion 3. (AE) NOESY-1D (CD3OD, 500 MHz). (AF) Key COSY, HSQC-TOCSY and HMBC correlations. (AG) Relative configuration proposed for disaccharide moiety. (AH) UV and optical rotation $[\alpha]_D^{23}$ = - 96.4 (c 0.21, MeOH); UV (MeOH) λ_{max} (log ε) 204 (4.8), 254 (4.7), 334 (3.8); 1H and 13C NMR (CD3OD, 500/125 MHz); HRESIMS m/z 1463.8282 [M + H] + (calculated for C77H123O26, 1463.8297, err 1.0 ppm). (AI) 1H and 13C NMR (CD3OD, 500/125 MHz).

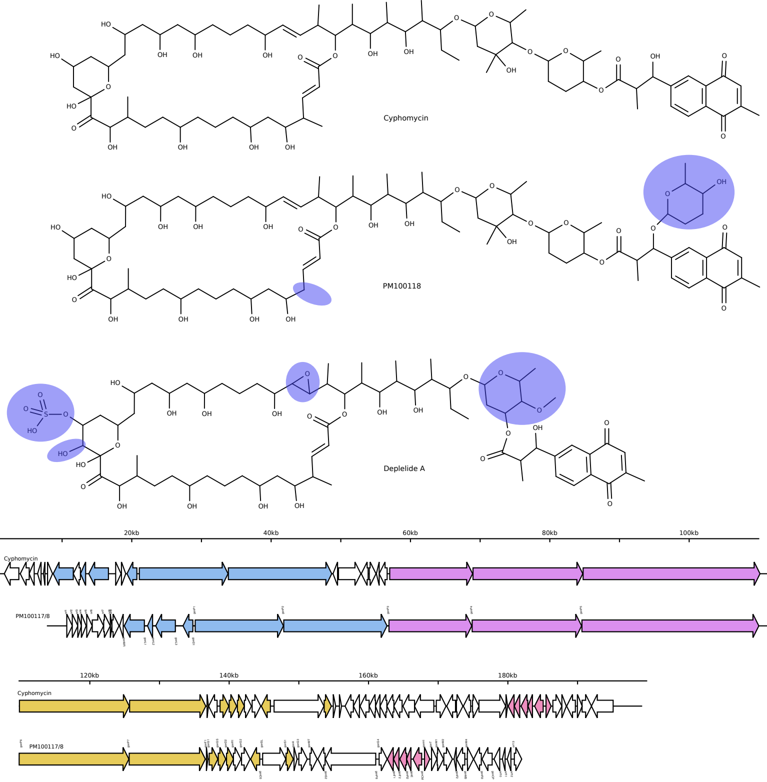

Supplementary Figure 4. Proposed biosynthetic logic of cyphomycin. The structures of cyphomycin, PM100118, and deplelide A are shown with key differences marked in blue. Specifically, PM100117 and PM100118 do not have a methyl on carbon 4. The second sugar in the PM100118 tail, L-2-deoxifucose, alternatively be hydroxylated and become L-rhodinose in PM100117. In contrast to cyphomycin, both have an L-rhodinose as the third sugar in the tail, the loss of which has been shown to greatly effect antitumor activity. Deplelide A varies from cyphomycin in many places, including an extra sulfonic acid, epoxide, and hydroxyl attached to the core macrolide, and variance in both the number and type of sugars in the tail. Cyphomycin has a β-amicetose in second sugar unit compared to PM100117/8. Also shown are the biosynthetic gene clusters for cyphomycin and PM100117/8. Here we propose a similar T1PKS biosynthetic logic for the macrolide core as that described previously for PM100117/8, from an isolate associated with a marine polychaete worm of the genus *Filograna*. Open reading frames are depicted as arrows and colors correspond to groups shared gene content between the two clusters (proteins have over 75% coverage and 75% identity). Genes that are not shared between the two are colored white. There are no differences in domain or predicted elongation unit specificity within the T1PKS itself. Interestingly, the acyltransferase of module 19 in PM100117/8 (first module of gonP7) is predicted to encode specificity for methylmalonyl-CoA, but the final structure is missing this methyl. This suggests either a malonyl-CoA is incorporated at this position or the methyl is cleaved in post-PKS tailoring. In contrast, cyphomycin has this methyl moiety.
