# Supplementary material for: The antimicrobial potential of Streptomyces from insect microbiomes
Source: Nat Commun. 2019 Jan 31;10:516. doi: 10.1038/s41467-019-08438-0 (PMC6355912; doi:10.1038/s41467-019-08438-0)
Supplement: Supplementary file 2 — Description of Additional Supplementary Files [file 41467_2019_8438_MOESM2_ESM.docx]

**Description of Supplementary Files**

**File Name:** Supplementary Data 1

**Description:** Host sampling and GPS coordinates.

**File Name:** Supplementary Data 2

**Description:** Summary of sampling by source.

**File Name:** Supplementary Data 3

**Description:** Summary of inhibition bioassays by pathogen.

**File Name:** Supplementary Data 4

**Description:** Complete inhibition bioassay dataset.

**File Name:** Supplementary Data 5

**Description:** Network of BGCs and BGC family identification using BiG-SCAPE.

**File Name:** Supplementary Data 6

**Description:** Untargeted LC/MS metabolomics of Streptomyces.
